# Endothelial injury and acute-phase inflammatory mediators converge to drive dengue severity

**DOI:** 10.3389/fimmu.2026.1970274

**Published:** 2026-10-08

**Authors:** Abdul R. Anshad, Muthuvel Atchaya, Shanmugam Saravanan, Amudhan Murugesan, Pachamuthu Balakrishnan, Sivadoss Raju, Yean K. Yong, Marie Larsson, Esaki M. Shankar

**Affiliations:** 1 Infection and Inflammation, Department of Biotechnology, Central University of Tamil Nadu, Neelakudi, Thiruvarur, India; 2 Dental Research Cell, Dr. D. Y. Patil Dental College and Hospital, Dr. D. Y. Patil Vidyapeeth, Pune, Maharashtra, India; 3 Deparment of Microbiology, The Government Theni Medical College and Hospital, Theni, India; 4 Department of Research, Meenakshi Academy of Higher Education and Research, Chennai, India; 5 State Public Health Laboratory, Directorate of Public Health and Preventive Medicine, Chennai, India; 6 Laboratory Center, Xiamen University Malaysia, Sepang, Malaysia; 7 Molecular Medicine and Virology, Department of Biomedical and Clinical Sciences, Linköping University, Linköping, Sweden

**Keywords:** angiopoietin, chymase, CRP, dengue, endothelial dysfunction

## Abstract

**Introduction:**

Severe dengue infection is characterized by endothelial injury and systemic inflammatory complications. To better understand the mechanisms underlying disease severity, we investigated a broad panel of circulating inflammatory and endothelial mediators in patients with clinical dengue infection.

**Methods:**

A prospective cross-sectional study was carried out involving 111 dengue patients and 42 healthy controls. Among the dengue cases, 85 were identified as primary, while 26 were classified as secondary dengue infections. Serum levels of endothelial markers (Ang-2, CXCL10, MCP1, and TRAIL), liver dysfunction and acute-phase markers (CRP, galectin 3, and serum amyloid protein), systemic inflammatory mediators (MIF, TNF-α, IL-1β), mast cell-derived proteases (chymase, tryptase), and tissue repair markers HGF, IL-10, IL-1Ra) were quantified using ELISA and Luminex multiplex assays. Correlations among serum analytes, severity indicators, and haematological markers were also explored.

**Results:**

Several biomarkers, Ang-2, CXCL10, TRAIL, CRP, MIF, IL-1Ra, TNF-α, and chymase showed differential expression across severity groups, indicating coordinated endothelial and inflammatory activation. Stratification of patients with primary-secondary dengue also followed a similar pattern except IL-1β, which had significant differential expression across the cohorts. Ang‐2 showed strong positive correlations with markers of hepatic dysfunction, including ALT, AST, and bilirubin, suggesting a link between endothelial injury and liver involvement.

**Conclusions:**

Severe dengue is driven by the coordinated activation of endothelial dysfunction, acute-phase responses, mast cell mediators, and counter-regulatory pathways. These processes collectively contribute to vascular leakage and organ injury, reinforcing the value of biomarkers such as Ang-2, CXCL10, CRP, and chymase for severity assessment.

## 1 Introduction

Dengue virus (DENV), a member of the *Flaviviridae* family, transmitted by female *Aedes aegypti* and *Aedes albopictus*, infects ~400 million people annually and remains hyperendemic in many tropical and sub-tropical regions (1–3). With expanding mosquito geographical distribution, driven in part by anthropogenic activities and global temperature changes, dengue distribution appears to expand beyond endemic regions where it is less reported otherwise (4, 5). The increasing incidence of dengue, coupled with diverse clinical presentations across serotypes and primary versus secondary infections, underscores the need for improved diagnostic and prognostic tools, especially to predict dengue severity (6). Based on the clinical features and disease progression, dengue is classified into three primary categories: dengue without warning signs (DWS-), dengue with warning signs (DWS+), and severe dengue (7). Dengue disease progression is influenced by a combination of immune dysregulation and direct viral effects, and the specific pathomechanisms remain inscrutable (8). Clinical presentation ranges from subclinical/asymptomatic dengue to fatal disease encompassing severe thrombocytopenia and hypovolemic shock, as well as multi-organ impairment (9). Severe complications of the multi-organ impairment could culminate in poor prognosis e.g., vascular derangement, renal dysfunction, dengue meningitis, and hepatic encephalopathy (10, 11).

Endothelial dysfunction represents the central axis of dengue pathogenesis and is a key driver of severe disease contributing to thrombocytopenia and dengue-associated shock (12, 13). Mediators such as angiopoietin 2 (Ang-2), CXCL10 (C-X-C motif chemokine ligand 10), CCL2(C-C motif chemokine ligand 2)/MCP-1(Monocyte chemoattractant protein-1), TNFα (tumor necrosis factor alpha), IL-1β, and TRAIL (TNF-related apoptosis inducing ligand)TNFSF-10 reflect endothelial activation, leukocyte recruitment, and apoptosis, which collectively contribute to increased vascular permeability and capillary leakage by destabilising the endothelial junction (14, 15). Hepatic involvement represents another prominent feature of dengue infection, ranging from mild transaminitis to acute liver dysfunction (16). The DENV induced hepatic-immune modulation can be measured using acute-phase proteins, C-reactive protein (CRP), and serum amyloid protein (SAP), as well as immune markers, including galectin 3, macrophage migratory inhibitory factor (MIF), IL-10, TNFα, IL-1β, and CXCL10. Together these markers indicate both hepatic dysfunction and the broader systemic dengue disease burden (17–20).

Aside from hepatic involvement, pulmonary and renal disorders are also increasingly recognised in clinical dengue infection (21, 22). Pulmonary disorders including acute lung injury or acute respiratory distress syndrome (ARDS) are closely associated with increased endothelial permeability (23). Further, DENV-mediated mast cell activation appears to be a determinant of clinical dengue as measured by the levels of chymase, tryptase, and TNF-α, which are indicative of capillary permeability and acute-phase inflammatory responses. In contrast, the tissue repair as well as counter-regulatory pathways in clinical dengue is often reflected by the relative concentrations of hepatocyte growth factor (HGF) and IL-1 receptor antagonist (IL-1RA), which affirms the host’s attempt to resolve inflammation during the recovery phase to restore endothelial integrity. In this study, we investigated a broad range of systemic mediators of inflammation and endothelial dysfunction to underpin their potential associations with disease severity in dengue infection. Here, we hypothesised that increasing disease severity could be associated with coordinated dysregulation of endothelial, inflammatory and immune-regulation mediators, resulting in distinct circulatory immune landscape among patients with varying magnitudes of dengue severity.

## 2 Materials and methods

### 2.1 Ethics approval

The study was reviewed and approved by the Institutional Ethical Committee (IEC) of the SMCH, Chennai (Ref. No. 114/03/2024/Faculty/SRB/SMCH) and TMC, Theni (Ref. No. 2300/IEC/2024-26), and was carried out in accordance with the Guidelines of the International Conference on Harmonization Guidelines and the Declaration of Helsinki (1964). All the human subjects were adults and provided written consents to participate in the study. Blood specimens were collected in BD Vacutainer tubes (Becton Dickinson, Franklin Lakes, NJ, USA) during the distinct phases of dengue infection, between 3 and 5 days after onset of symptoms/signs by a trained phlebotomist and were stored at -80 °C until use in the experiments (in 100 µL aliquots to minimise multiple freeze-thaw cycles).

### 2.2 Study population

DENV-infected individuals were recruited prospectively based on clinical symptoms, and were further confirmed by dengue-NS1 antigen or anti-dengue IgM ELISA (1, 24). The NS1 antigen test was performed at the time of blood collection by the clinicians using a commercial NS1 ELISA kit (cat. no. 01PE40, Abbott, Chicago, IL, USA). PanBio dengue IgM (cat. no. 01PE20, Abbot, USA) and PanBio dengue IgG (cat. No. 01PE10, Abbot, USA) kits were used to classify the participants/patients into primary and secondary dengue. Samples with positivity in both ELISAs with an IgM/IgG of ≤1.2 were considered secondary dengue, whereas IgM^+^IgG^+^ samples with an IgM/IgG ratio >1.2 or IgM^+^IgG^-^ were categorized as primary dengue.

### 2.3 Case-definition

Dengue severity was assessed based on the classification into either of the following: dengue with warning signs (DWS+), dengue without warning signs (DWS-), and severe dengue based on the clinical and laboratory findings recorded during specimen collection. DWS- was defined as dengue infection without any warning signs or criteria for severe dengue, while DWS+ was defined as the presence of one or more WHO-defined warning signs, including retro-orbital pain, conjunctival suffusion, abdominal pain, persistent vomiting, arthralgia, clinical fluid accumulation, mucosal hemorrhage, restlessness, hepatomegaly, thrombocytopenia, and hepatomegaly. Severe dengue classification was based on severe plasma leakage leading to shock, severe bleeding, and/or severe organ dysfunction (25).

### 2.4 Luminex multiplex assay

Serum concentrations of chemokines and cytokines were quantified by a commercial Bio-Plex Luminex multiplex assay (Bio-Rad Laboratories, Hercules, CA, USA) with the Human Luminex Discovery 9-plex (cat. no. LXSAHM-09, R&D Systems, Minneapolis, USA). The analytes measured were CCL2/JE/MCP-1, HGF (hepatocyte growth factor), IL-1Ra/IL-1F3, MIF, TRAIL (TNF-related apoptosis-inducing ligand)/TNFSF-10, CXCL10/IP-10/CRG-2/IL-1β/IL-1F2, IL-10, and TNF-α. Frozen serum stored in batches were thawed at 4 °C before dilution at a 1:4 ratio using sample diluent and analyzed on a Bio-Plex 200 System according to the manufacturer’s instructions. The IL-1Ra/IL-1β ratio was calculated to represent the relative balance between the anti-inflammatory IL-1Ra and pro-inflammatory IL-1β, in order to provide an integrated measure of inflammatory and counter-regulatory responses across the clinical groups.

### 2.5 Enzyme-linked immunoassays

Commercial ELISAs were employed for measuring circulating liver dysfunction and acute-phase mediators such as serum amyloid P (SAP) (cat. no. ab246537, Abcam, Cambridge, UK; sensitivity 181pg/ml), nephritic marker galectin-3 (cat. no. ab269555, Abcam, Cambridge, UK; sensitivity 13.3pg/ml), and C-reactive protein (CRP) (cat. no. DCRP00B, Biotechne, Minneapolis, USA; sensitivity 0.022ng/ml), were quantified as per the manufacturer’s instructions. The endothelial activation marker, angiopoietin-2 (Ang-2) was also measured using a commercial ELISA (cat. no. DANG20, Biotechne, Minneapolis, USA sensitivity 21.3pg/ml). Besides, the mast cell-specific proteases, tryptase (cat. no. ab314709, Abcam, Cambridge, UK; sensitivity 2ng/ml) and chymase (cat. no. CSB-E13757h, Cusabio, Houston, USA; sensitivity 0.039ng/ml), were also measured by commercial ELISA.

### 2.6 Statistical analysis

All categorical variables were presented as number (percentage) and all continuous variables were presented in median (interquatile range). Categorical variables were analysed using Chi Square test whereas all continuous variables were analysed using Kruskal–Wallis’s test followed by Mann–Whitney U-tests *post-hoc* tests to compare the levels of each biomarker across the DWS-, DWS+, and the severe dengue groups as well as the primary and secondary dengue cohorts. Logistic regression models were constructed to assess the associations between specific biomarkers and the risk of severe disease. Correlation analysis was performed to determine the relationship between immune markers and clinical severity scores. Statistical significance was set at a P value <0.05 for all the analyses. This comprehensive methodology allowed for the examination of immune dysregulation, vascular involvement, and renal impairment markers in dengue patients. By profiling these markers across different severity levels, we aimed to elucidate the molecular and immunological drivers of severe dengue, with a focus on identifying biomarkers that could guide early diagnosis and targeted intervention.

## 3 Results

### 3.1 Clinico-demographic data of participants recruited in the dengue investigation

In this prospective cross-sectional study, we recruited 153 participants, which included 111 dengue patients (72.54%) encompassing primary (n=85; 76.57%) and secondary (n=26; 23.42%) infections, and healthy controls (n=42; 27.45%). In the dengue cohort, 35 participants were positive for anti-DENV IgG, while 103 were positive for anti-DENV IgM. Twenty-four subjects were positive for NS1, of which 18 tested positive for both IgM and NS1 (**Table 1**). All participants in the study were adults, aged over 18 years. The workflow of the investigation is illustrated in **Figure 1**. Further, of the 111 patients included, 48 (43.24%), 55 (49.54%) and 8 (7.20%) were classified as those with DWS+, DWS-, and severe dengue, respectively.

**Table 1:** Cohort characteristics for demography and laboratory characters at admission.

Demographic and laboratory parameters	Total	Healthy controls	Dengue patients
**Age,** in years	33.35	27.92	35.44
**Sex,** male, *n (%)*	111 (72.54)	42 (100)	69 (62.16)
**Dengue category**			
DWS- *(%)*	--	--	48 (43.24)
DWS+ *(%)*	--	--	55 (49.55)
severe dengue *(%)*	--	--	8 (7.21)
**IgM positive,** *n (%)*	--	--	103 (92.79)
**IgG positive,** *n (%)*	--	--	35 (31.53)
**1° infection,** *n (%)*	--	--	85 (76.58)
**2° infection,** *n (%)*	--	--	26 (23.42)
**NS1 positive**, *n (%)*	--	--	24 (21.62)

Patients’ characteristics for demography, laboratory characteristics at admission. Comparison of demographic and clinical characteristics between dengue patients and healthy control. Percentages are given in the bracket.

Age, expressed in years; Sex, presented as the number and percentage of male participants; Dengue category, classified as dengue without warning signs (DWS-), dengue with warning signs (DWS+), and severe dengue (SD); IgM positive, participants positive for DENV-specific immunoglobulin M; IgG positive, participants positive for DENV-specific immunoglobulin G; 1° infection, primary dengue infection; 2° infection, secondary dengue infection; and NS1 positive, participants positive for the non-structural protein 1 antigen of DENV.

**Figure 1:**
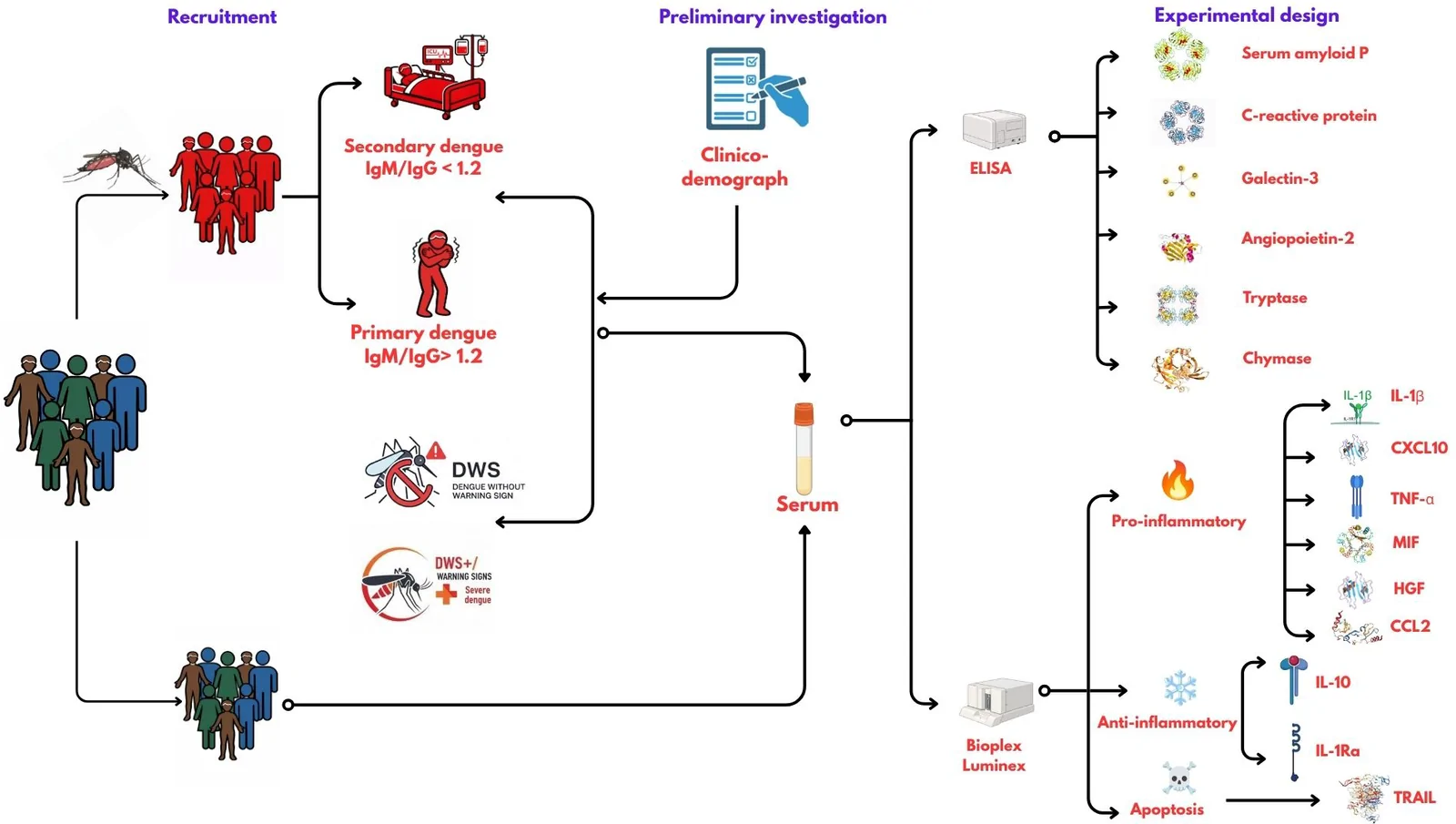
Graphical abstract of the cross-sectional case-control investigation of organ dysfunction markers as well as cytokines. The cohort was divided in to dengue with warning signs, dengue without warning signs, severe dengue which also stratified in another cohort of primary and secondary dengue based on WHO 2007 classification. The serum samples were collected from adult patients/volunteers with proper consent and were measured using ELISA as well as Bioplex Luminex cytokine array systems. CCL2, C-C motif chemokine ligand 2; CXCL10, C-X-C motif chemokine ligand 10; DWS-, dengue without warning signs; DWS+, dengue with warning signs; ELISA, enzyme-linked immunosorbent assay; HGF, hepatocyte growth factor; IgG, immunoglobulin G; IgM, immunoglobulin M; IL-1β, interleukin-1 beta; IL-1Ra, interleukin-1 receptor antagonist; IL-10, interleukin-10; MIF, macrophage migration inhibitory factor; TNF-α, tumor necrosis factor-alpha; TRAIL, TNF-related apoptosis-inducing ligand.

### 3.2 Mediators linked to endothelial dysfunction and capillary permeability markers were significantly elevated in dengue with warning signs and severe dengue

Mediators of endothelial dysfunction and vascular permeability were significantly elevated in DWS+ and in those with severe disease. CXCL10 levels were markedly higher in all dengue groups relative to the healthy controls, with the highest increase observed among DWS+ (P<0.0001) and severe dengue (P<0.0001) groups (**Figure 2**). Ang-2 also showed a progressive increase, with the highest levels observed in the severe dengue (P<0.05) as well as DWS+ (P<0.05) groups than in the DWS- cohort (P<0.001), while remaining lower in the healthy cohort (**Figure 3**). CCL2/MCP-1 (**Figure 2**) levels were moderately elevated in the dengue cohorts, although not significantly so. In contrast, TRAIL/TNFSF-10 levels (**Figure 2**) were increased in dengue patients with a higher elevation observed across both DWS+ (P<0.05) and severe dengue (P<0.05) patients than the DWS- patients (P<0.01). Overall, these findings indicated that inflammatory mediators associated with endothelial activation and capillary permeability were markedly elevated in severe dengue infection.

**Figure 2:**
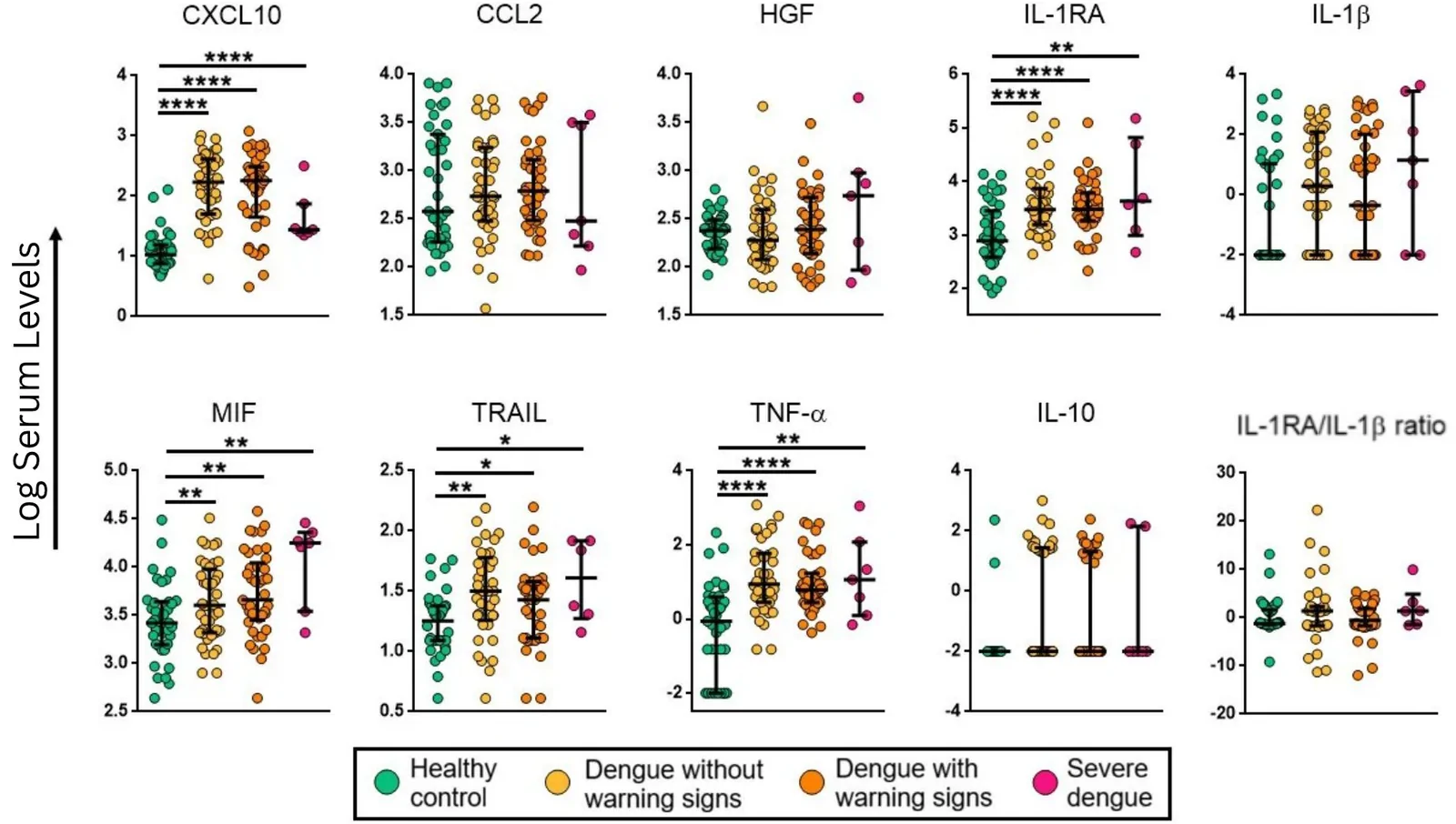
Comparison of serum levels of cytokines in healthy controls, dengue without warning signs, dengue with warning signs and severe dengue compared with healthy controls. Levels of biomarkers were compared across the four study groups by the Kruskal–Wallis test. *Post hoc* Mann–Whitney U-tests were subsequently performed for those biomarkers with a Kruskal–Wallis P-value of <0.05. P-value <0.05 (significant); *, <0.05; **, <0.01; ***, <0.001, ****, <0.0001. CCL2, C-C motif chemokine ligand 2; CXCL10, C-X-C motif chemokine ligand 10; HGF, hepatocyte growth factor; IL-1β, interleukin-1 beta; IL-1Ra, interleukin-1 receptor antagonist; IL-10, interleukin-10; MIF, macrophage migration inhibitory factor; TNF-α, tumor necrosis factor-alpha.

**Figure 3:**
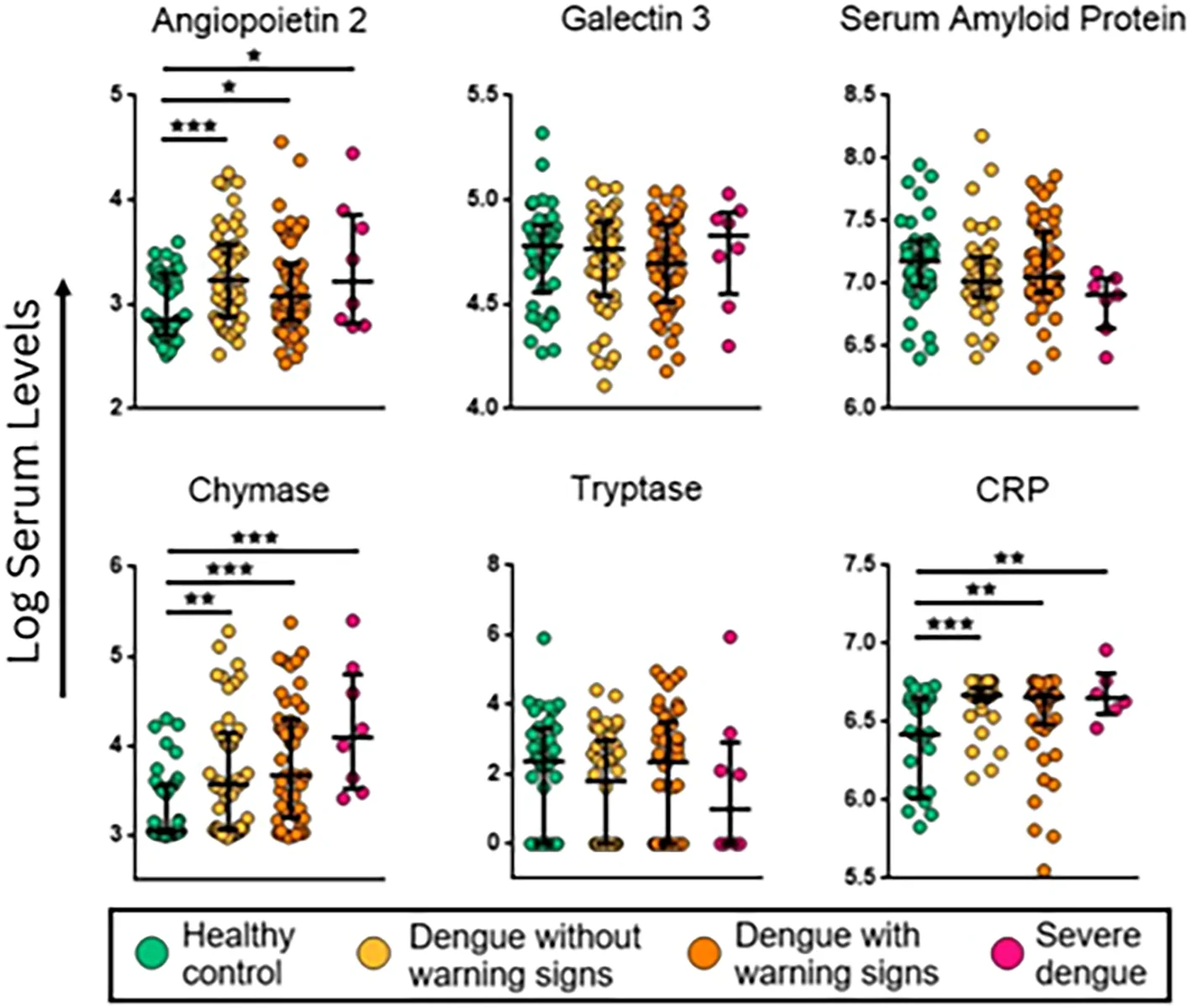
Comparison of serum levels of organ dysfunction analytes in dengue without warning signs, dengue with warning signs and severe dengue compared with healthy controls. Levels of biomarkers were compared across the four study groups by Kruskal–Wallis test. *Post hoc* Mann– Whitney U-tests were subsequently performed for those biomarkers with a Kruskal–Wallis P-value of <0.05. P-value <0.05 (significant); *, <0.05; **, <0.01; ***, <0.001, ****, <0.0001. CRP, C-reactive protein.

### 3.3 Patients with dengue with warning signs displayed a relative systemic abundance of mediator’s suggestive of inflammation and hypercytokinemia

We next measured MIF levels (**Figure 2**) across the cohorts, which were significantly elevated among dengue patients as compared to healthy controls, with the highest increase detected in the DWS+ patients (P<0.01). Besides, TNF-α and IL-1β were also found to be elevated among dengue patients compared to healthy controls (**Figure 2**). Of note, TNF-α levels were significantly elevated among DWS+ (P<0.0001) as well as severe dengue (P<0.01) patients but IL-1β showed a non-significant elevation across the group. Taken together, the findings indicate heightened inflammatory response consistent with acute inflammation and cytokine storm in clinical dengue infection.

### 3.4 CRP levels showed significant increase whereas SAP and galectin 3 concentrations were not markedly different across the case-control cohorts

Having measured the levels of endogenous pyrogens TNF-α and IL-1β that are activators of production of acute-phase reactants by the liver, next, we determined the relative levels of mediators associated with hepatic injury and acute-phase reactants (often released by the liver) such as CRP, SAP and galectin 3. Our findings demonstrated that the levels of CRP (**Figure 3**) were significantly elevated in the DWS+ (P<0.01), severe dengue (P<0.01), and DWS- (P<0.001) cohorts compared to the healthy control cohort, while no statistical difference was observed in levels of CRP between the dengue cohorts. Meanwhile, the levels of serum amyloid protein (**Figure 3**) and galectin 3 (**Figure 3**) did not show any significant alterations across the different case-control cohorts.

### 3.5 Serum chymase levels were significantly elevated indicative of potential activation of mast cells in clinical dengue disease

The levels of chymase and tryptase, key markers of mast cell activation, were next assessed. Systemic chymase concentrations were significantly elevated both in the severe dengue (P<0.001) as well as the DWS+ (P<0.001) cohorts compared to the DWS- (P<0.01) and healthy control groups. In contrast, serum tryptase levels (**Figure 3**) were varied with modest elevation observed among dengue patients but demonstrated a non-significant difference across the group. These findings suggest activation of mast cells during clinical dengue infection.

### 3.6 Heightened levels of mediators associated with tissue repair and counter regulatory responses were evident in clinical dengue infection

We next examined levels of mediators involved in tissue repair and counter regulatory responses in clinical dengue infection. HGF levels (**Figure 2**) were modestly increased in dengue patients compared with controls, with slightly higher levels in severe dengue although there was no significant difference between the groups. In contrast, IL-1Ra (**Figure 2**) concentrations were significantly increased across the dengue groups, particularly in severe dengue (P<0.01) and DWS+ (P<0.0001). These findings indicate activation of pathways associated with tissue repair and counter-regulatory responses in dengue infection. Similarly, IL‐10 levels did not show significant variation between the cohorts. The IL-1Ra/IL-1β ratio also did not show a statistical difference between the clinical cohorts.

### 3.7 Broad elevation of inflammatory, endothelial, and mast cell mediators was observed in dengue infection across both primary and secondary cohorts

Next, we sought to compare the analytes between the primary and secondary dengue cohorts. Most of the analytes followed patterns similar to the earlier cohort stratification, albeit with a few notable changes. Of the endothelial biomarkers, CXCL10, TRAIL and Ang-2 showed significant differential expression across the cohorts, while CCL2 expression remained non-significant across the groups. CXCL10 expression was elevated in both primary (P<0.0001) and secondary dengue (p<0.0001) compared to the healthy controls. Ang-2 was significantly higher in primary dengue compared to healthy controls (P<0.001), and TRAIL levels were increased in both primary (P<0.05) and secondary dengue (P<0.05) as compared to healthy controls.

MIF, TNF-α, and IL-1β showed significant alterations across the cohorts, with MIF showing a significantly higher expression in primary dengue than in healthy controls (P<0.01), while TNF-α and IL-1β levels were significantly higher in both primary (TNF-α, P<0.0001; IL-1β, P<0.05) and secondary dengue (TNF-α, P<0.001; IL-1β, P<0.05) cohorts. SAP and galectin 3 showed non-significant alterations between the cohorts, while the expression of CRP was higher in primary (P<0.001) and secondary dengue (P<0.001) relative to the healthy controls. The levels of IL-1Ra were significantly increased in primary (P<0.0001) and secondary (P< 0.01) dengue. Likewise, the expression of mast cell-derived analytes also showed a similar pattern as in the earlier cohort stratifications as DWS+ and DWS-. Chymase levels were higher across both primary (P<0.01) and secondary dengue (P<0.0001) than in healthy controls. Moreover, chymase levels in secondary dengue were significantly higher than in primary dengue (P<0.01), while tryptase concentrations remained non-significant across the cohorts (**Figures 4**, **5**).

**Figure 4:**
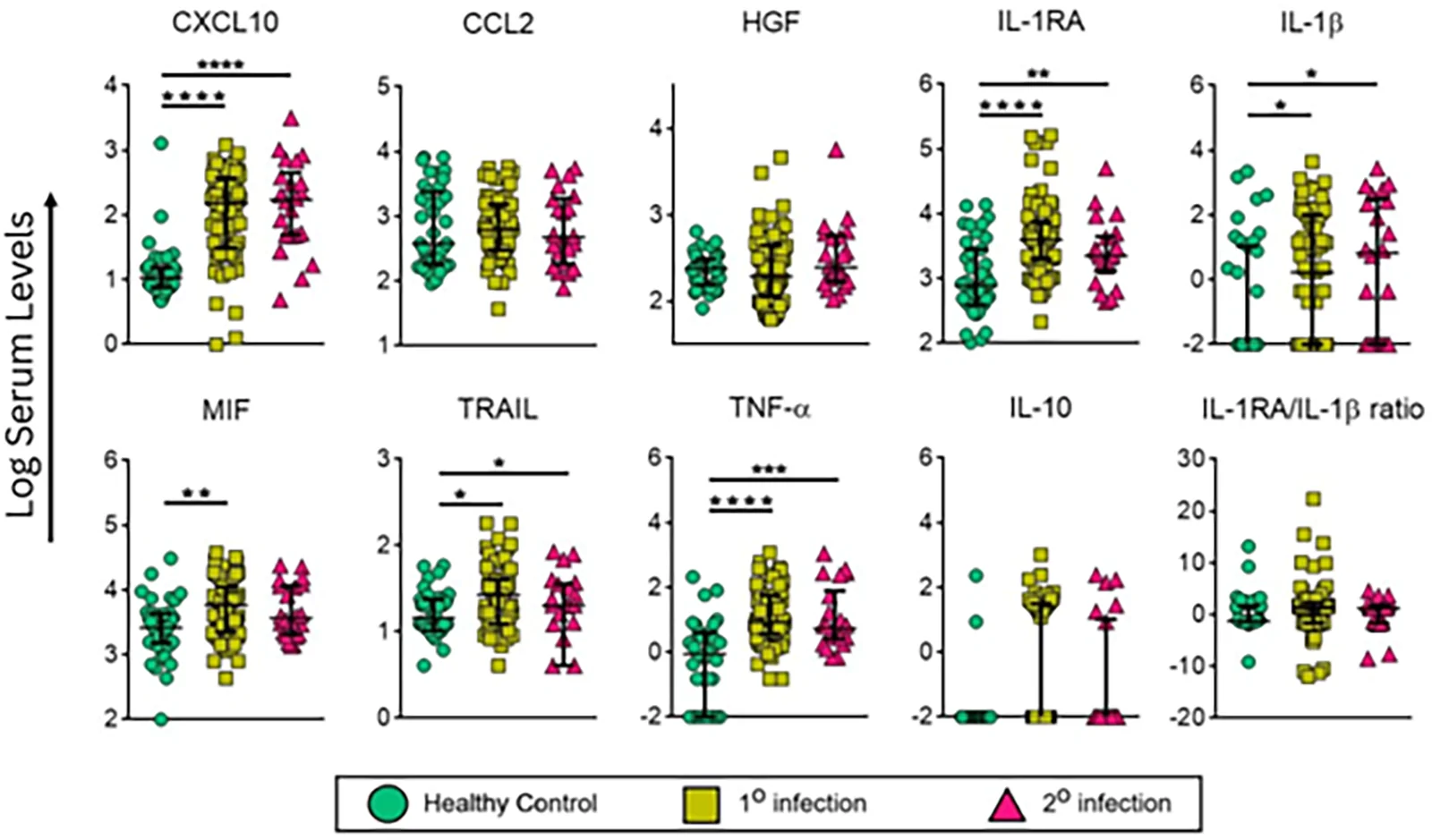
Comparison of serum levels of cytokines analytes in primary and secondary dengue with healthy controls. Levels of biomarkers were compared across the three study groups by Kruskal–Wallis test. *Post hoc* Mann– Whitney U-tests were subsequently performed for those biomarkers with a Kruskal–Wallis P-value of <0.05. P-value <0.05 (significant); *, <0.05; **, <0.01; ***, <0.001, ****, <0.0001. 1°, primary; 2°, secondary, CCL2, C-C motif chemokine ligand 2; CXCL10, C-X-C motif chemokine ligand 10; HGF, hepatocyte growth factor; IL-1β, interleukin-1 beta; IL-1Ra, interleukin-1 receptor antagonist; IL-10, interleukin-10; MIF, macrophage migration inhibitory factor; TNF-α, tumor necrosis factor-alpha.

**Figure 5:**
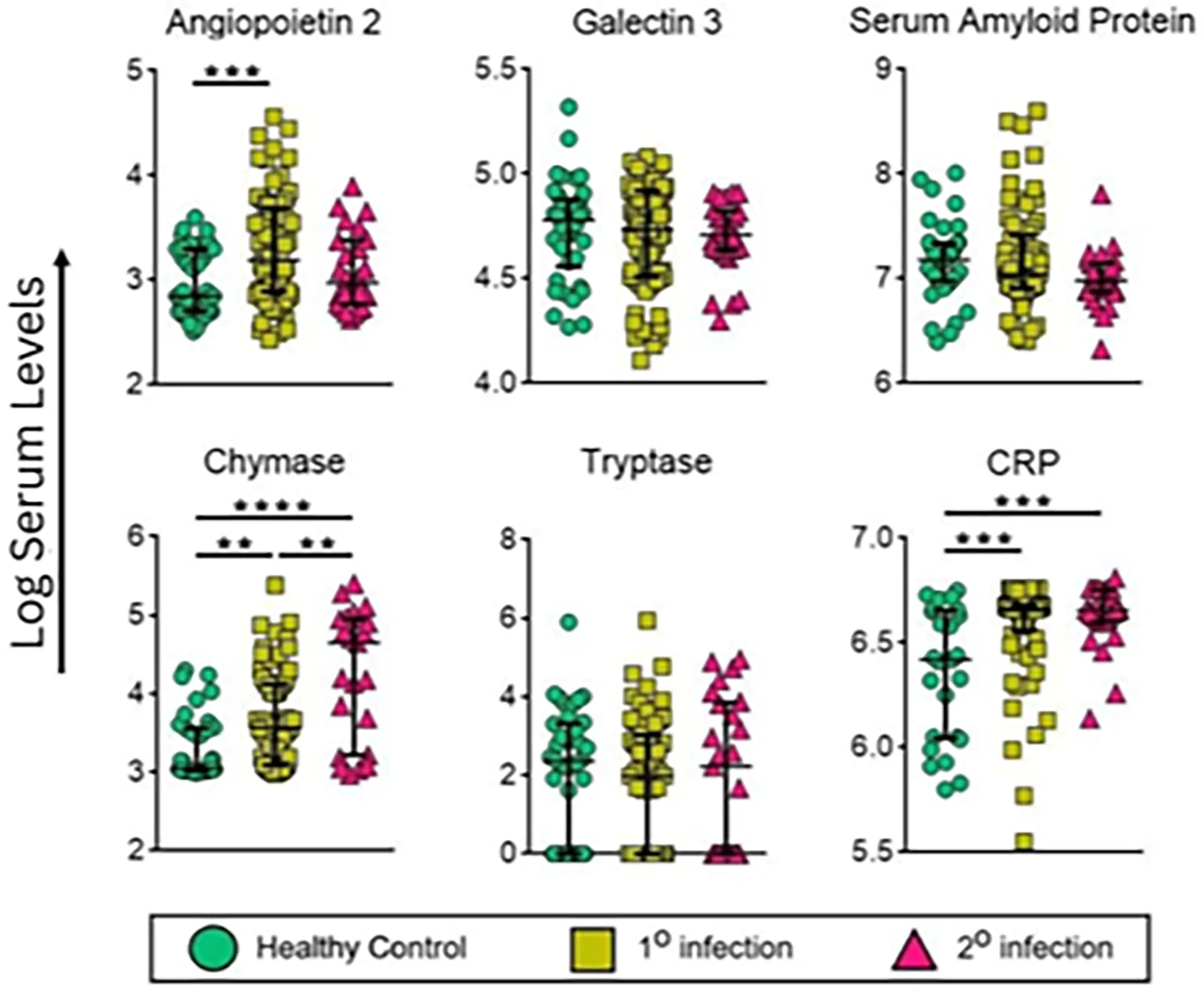
Comparison of serum levels of organ dysfunction analytes in primary and secondary dengue with healthy controls. Levels of biomarkers were compared across the three study groups by Kruskal–Wallis test. *Post hoc* Mann– Whitney U-tests were subsequently performed for those biomarkers with a Kruskal–Wallis P-value of <0.05. P-value <0.05 (significant); *, <0.05; **, <0.01; ***, <0.001, ****, <0.0001. 1°, primary; 2°, secondary; CRP, C-reactive protein.

### 3.8 Regression analysis revealed a strong positive association between disease severity and dengue progression

Next, we sought to perform a logistic regression analysis involving the different determinants of dengue severity, especially platelet counts and mediators and biomarkers of endothelial dysfunction. Initially, a logistic regression analysis was performed univariately to identify biosignatures that were significantly associated with dengue severity, platelet count, Ang-2, galectin 3 and SAP (**Figure 6A**). The mediators/biomarkers that showed a significant association in the univariate analyses were regarded as candidate predictors and were further included for analysis by a multivariate regression. Our findings showed that Ang-2 demonstrated a strong positive correlation between severity and several disease progression indicators, such as markers associated with thrombocytopenia and hepatic dysfunction i.e., liver enzyme and bilirubin levels. Galectin 3 showed limited association with most clinical parameters, while SAP demonstrated modest positive association with inflammatory markers but showed minimal association with classical laboratory indicators of organ dysfunction (**Figure 6A**). Of the predictors of disease severity, ALT and GGT were positively associated with severity, while RDW showed a negative association. While, erythrocyte counts showed a negative association with platelet levels, cholesterol levels were positively associated with platelet counts (**Figure 6B**).

**Figure 6:**
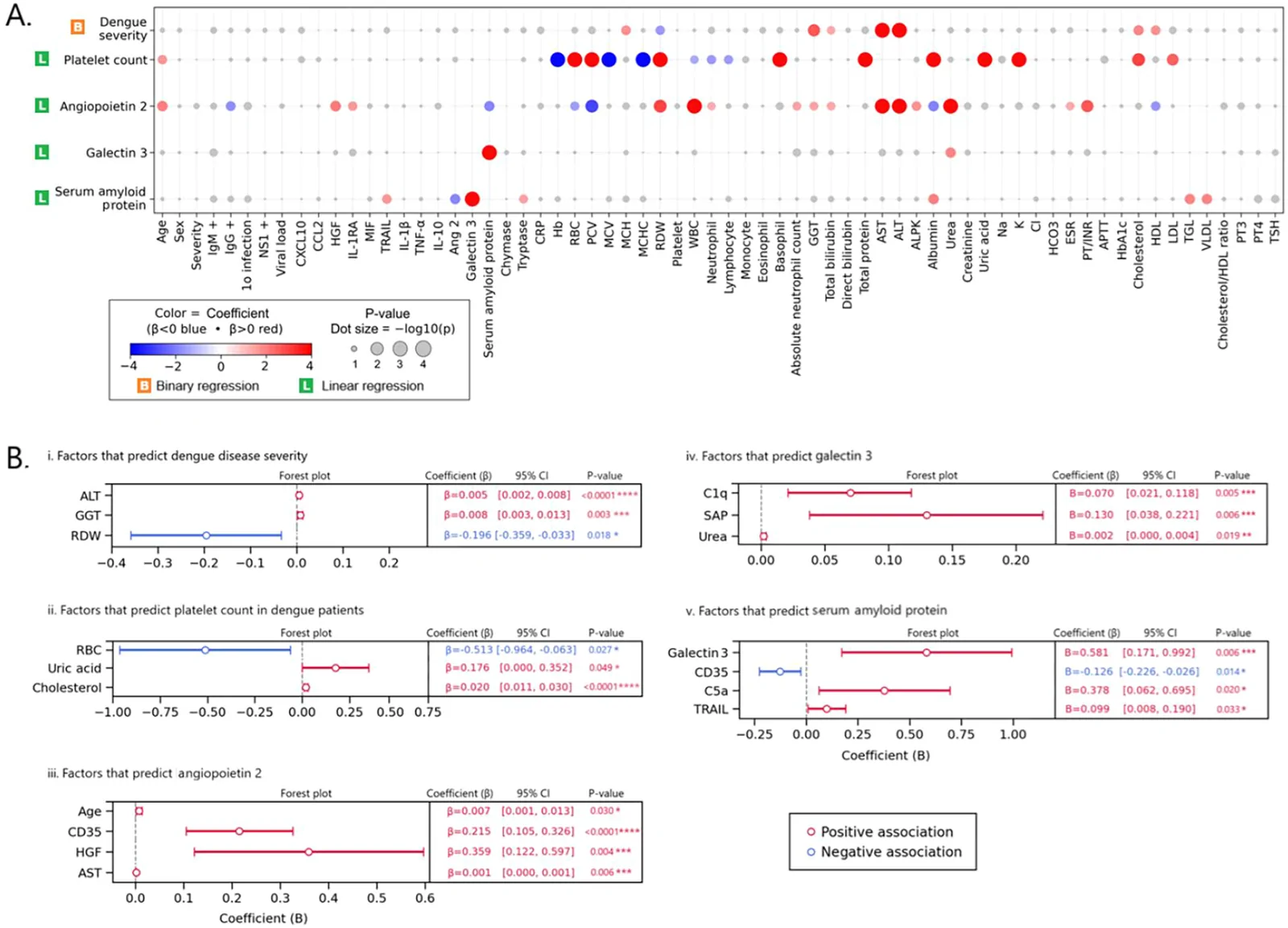
Factors associated with dengue severity, platelet counts and markers of endothelial dysfunction. **(A)** Heatmap showing the associations between dengue severity, platelet count, angiopoietin-2 (Ang-2), galectin-3, serum amyloid protein (SAP), and clinical, hematological, biochemical, and immunological parameters in dengue patients. Each dot represents the regression coefficient (β) describing the association between the predictor variable (x-axis) and the outcome variable (y-axis). Red dots indicate positive associations (β > 0), whereas blue dots indicate negative associations (β < 0). The colour intensity reflects the magnitude of the regression coefficient. Dot size is proportional to the statistical significance of the association, expressed as −log10 (P value), with larger dots indicating stronger statistical evidence. Grey dots represent non-significant associations. Dengue severity (DWS- versus DWS+ and severe dengue) was analyzed using binary logistic regression, while platelet count, Ang-2, galectin-3, and SAP were analyzed using linear regression. **(B)** Forest plot for multivariate analyses – where the variables identified as significant in the screening analysis (in **A**) were subsequently included in a stepwise multivariable regression models presented (in **B**), for which p values were <0.05, were considered as independent predictors. *, **, ***, and **** represent P values <0.05, <0.01, <0.001, and <0.0001, respectively. ALT, alanine aminotransferase; ALP, alkaline phosphatase; AST, aspartate aminotransferase; C1q, complement component 1q; C5a, complement component 5a; CCL2, C-C motif chemokine ligand 2; CRP, C-reactive protein; CXCL10, C-X-C motif chemokine ligand 10; ESR, erythrocyte sedimentation rate; GGT, gamma-glutamyl transferase; Hb, hemoglobin; HbA1c, glycated hemoglobin; HCO_3_^-^, bicarbonate; HDL, high-density lipoprotein; IgG, immunoglobulin G; IgM, immunoglobulin M; IL-1RA, interleukin-1 receptor antagonist; IL-10, interleukin-10; IL-18, interleukin-18; INR, international normalized ratio; LDL, low-density lipoprotein; MCH, mean corpuscular hemoglobin; MCHC, mean corpuscular hemoglobin concentration; MCV, mean corpuscular volume; Na, sodium; NS1, non-structural protein 1; PCV, packed cell volume; PT, prothrombin time; RBC, red blood cell; RDW, red cell distribution width; SAP, serum amyloid P protein; TG, triglycerides; TNF-α, tumor necrosis factor-alpha; TRAIL, TNF-related apoptosis-inducing ligand; TSH, thyroid-stimulating hormone; VLDL, very-low-density lipoprotein; WBC, white blood cell.

## 4 Discussion

Our prospective cross-sectional study demonstrated that dengue severity drives distinct alterations in the endothelial, inflammatory, mast cell-derived and counter-regulatory pathways, and concertedly promotes vascular permeability and organ dysfunction. These findings suggest the complex interplay between immune activation, endothelial injury, and regulatory response in dengue pathogenesis. However, the cross-sectional nature of the study means that the observed biomarker association should not be interpreted as evidence of direct mechanistic interaction or causality. Instead, these findings provide clinically relevant associations to generate hypotheses on key immunological pathways culminating in diverse clinical severity outcomes.

Among endothelial biomarkers, Ang‐2 demonstrated a clear severity‐dependent increase, with the highest levels observed among patients with warning signs and severe dengue (26). Ang-2 is a key antagonist ligand of the Tie2 pathway, which competitively inhibits Ang-1 binding. It is secreted in diseased vasculature and is implicated with angiogenesis and vascular stability (27). It promotes endothelial destabilization and vascular leakage, and is also a known predictor of mortality in critically ill individuals (28). In primary dengue, Ang-2 increase may reflect early endothelial activation via exocytosis of Weibel-Palade bodies. In primary dengue, the response is contingent upon naïve immunity without ADE-mediated Fc receptor, unlike severe secondary dengue, where Ang-2 emerges from viral-endothelial interactions or pro-inflammatory signals (29).

The surge in CXCL10 levels observed in DWS+ and severe dengue appears to hint its role in endothelial activation (13, 18). Further, increased CXCL10 could be linked to heightened increased dengue severity owing to exaggerated inflammation (18, 30, 31). Together with Ang-2 expression, this axis might be representative of dengue-associated inflammation and destabilisation of the endothelium entailing plasma leakage. Similarly, relative abundance of CXCL10 in primary and secondary dengue suggests an enhanced IFN-mediated antiviral response and immune cell recruitment (32, 33).

Increased CCL2 is primarily indicative of monocyte recruitment rather than an indication of dengue disease severity (34). In contrast, increased TRAIL levels in dengue may reflect its role in immune mediated cytotoxic activity and endothelial stress, although its precise role in vascular dysfunction needs to be explored further. Functional studies indicate that TRAIL could be antiviral in DENV infection (35, 36). The elevation of the TRAIL in the DWS+ and severe dengue also aligns with immune regulation observed by others (37).

Apart from endothelial dysfunction, elevated TNF-α and MIF further indicates the amplification of pro-inflammatory cascades in dengue. TNF-α is a well-established mediator of vascular leakage and activation, while MIF sustains inflammatory responses by counteracting glucocorticoid-mediated suppression. Hence, their elevation is consistent with the hyperinflammatory milieu contributing to endothelial dysfunction. IL-1Ra was also markedly increased in dengue, consistent with activation of a compensatory anti-inflammatory response, whereas IL-1β showed variable expression without significant differences across the cohorts. This imbalance between pro-inflammatory response and counter-inflammatory pathway might be a reflection of immune dysregulation rather than a mere cytokine storm (9, 34, 38). Unlike in our cohort based on dengue severity, IL‐1β displayed significant differences when comparing primary and secondary dengue, with higher levels in secondary infection correlating with fever onset and vascular stress (39).

CRP levels were markedly increased in dengue patients, particularly in DWS+ and severe dengue, indicating an amplified acute-phase response with hepatic involvement (40, 41). In contrast, the SAP, galectin-3 and HGF levels remaining unchanged, suggests a selective rather than a global activation of the acute phase and growth factor response (42, 43). The significant elevation in CRP in both primary and secondary dengue further reflects strong acute systemic inflammatory responses (44).

The robust severity-dependent elevation of chymase in dengue patients, especially in DWS+ and severe dengue patients, supports its role in mast cell activation (45, 46). Chymase is known to be involved in disruption of endothelial barriers, matrix remodelling and vascular permeability (45, 46). The parallel elevation of chymase as well as Ang-2-CXCL10 axis suggests a mast cell-endothelial interaction contributing to plasma leakage. In contrast, the low levels of tryptase, another mast cell-derived mediator is suggestive of a selective mast cell mediator release rather than global degranulation in dengue severity (47, 48). The concurrent elevations in chymase, Ang-2, and CXCL10 should not be considered as a direct evidence of mast cell-endothelial interaction rather as a potentially plausible axis, which warrants experimental validation.

The strong positive association of Ang-2 with liver enzymes and thrombocytopenic markers further supports the earlier observation of a strong linkage between endothelial injury and systemic organ involvement (49). Regression analysis identified certain factors associated with inflammation. Age, HGF, and AST were significantly associated with Ang-2 levels, suggesting a potential link between hepatic functions and endothelial dysfunction. SAP and urea were associated with galectin-3, suggesting interactions between the acute-phase reactants and metabolic factors. In addition, SAP levels were significantly associated with galectin-3, CD163, and TRAIL, highlighting their likely role in macrophage stimulation, complement activation and immune signalling (1). Further, the observed association between the hematological and biochemical parameters may provide an indication of severity-associated systemic alterations. The positive associations of ALT and GGT with disease severity might be a reflection of the greater involvement of the hepatic system, which is widely reported in acute infections. Similarly, the association of GGT with severity suggests a potential hepatobiliary dysfunction during severe infection. The inverse association of the erythrocyte and platelet counts observed could be an indicator of concurrent haematological alterations. Likewise, the positive association between cholesterol and platelet counts likely reflects alterations in lipid metabolism and lipid homeostasis. Moreover, the negative association of RDW and severity reflects a disease- or cohort-specific variation warranting further investigation (1, 9, 12).

We have illustrated the sequence of events associated with dengue severity in **Figure 7**. Following DENV entry into the host, primary viral recognition is rendered by antigen-presenting cells triggering widespread immune activation, inflammatory cell recruitment, and cytokinemia, together with mast cell degranulation and release of DAMPs (alarmins). Vasoactive mediators in concert with pro-inflammatory cytokines (TNF-α and MIF) and chemokine signals (CXCL10), promote acute-phase responses (CRP, SAP and galectin-3) culminating in inflammation and capillary permeability rendering leukocyte transmigration into the interstitial tissue spaces. Contemporaneously, acute-phase responses in the liver and systemic compartments drive organ dysfunction and thrombocytopenia. To offset severe damage, counter-regulatory and repair signals (such as IL-1Ra and HGF) are mobilised. Together, the intersecting physiological cascades converge into pathological vascular leakage and multi-organ damage, the hallmark of clinical dengue pathogenesis.

**Figure 7:**
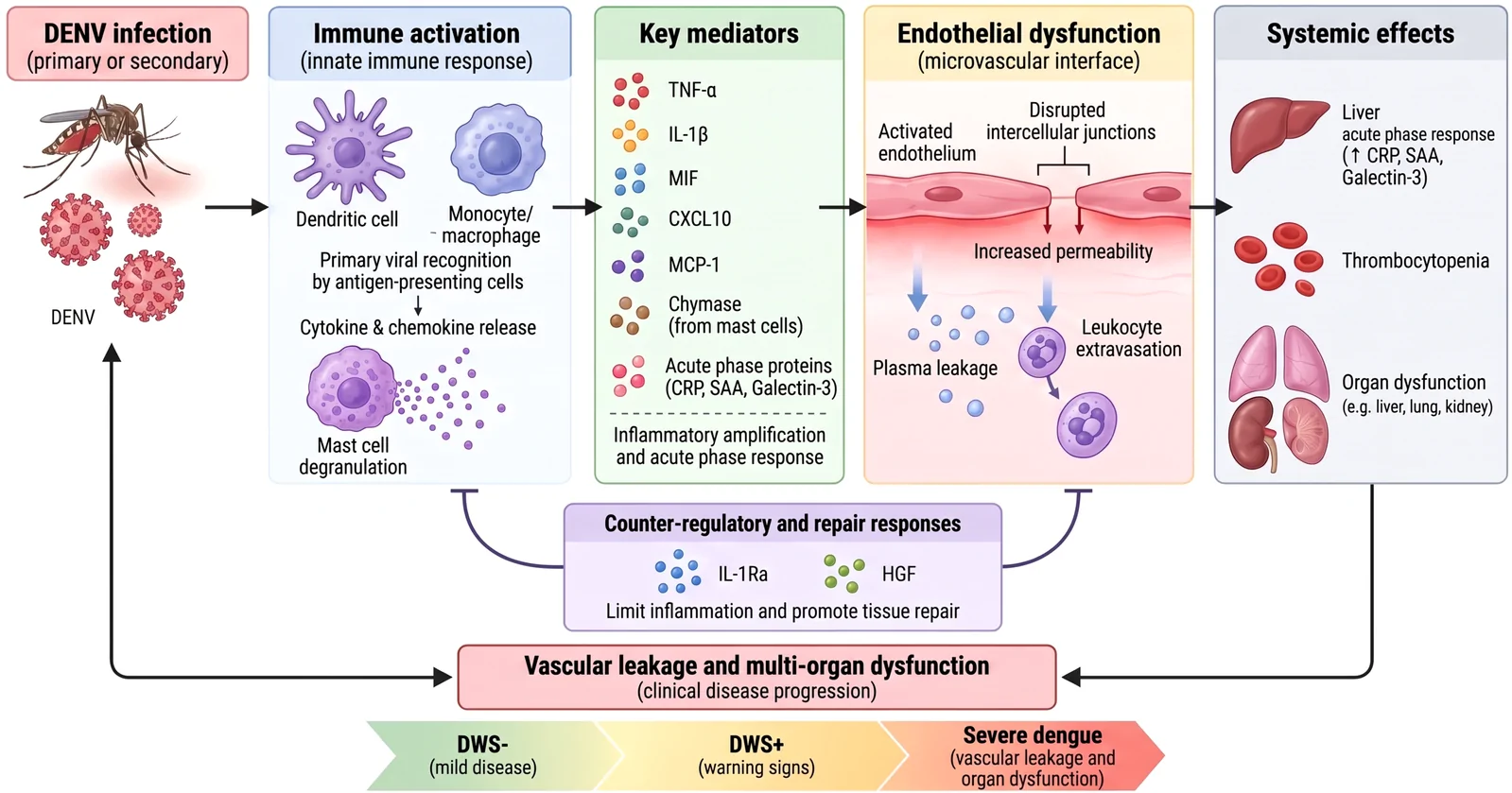
Proposed mechanistic pathway of dengue severity. The schematic depicts a mechanistic model of dengue pathogenesis, driving disease progression from primary or secondary dengue virus (DENV) infection to severe clinical outcomes. Following initial DENV infection, primary viral recognition by antigen-presenting cells (dendritic cells and monocytes/macrophages) triggers widespread immune activation, inflammatory cell recruitment, and cytokine release, alongside mast-cell degranulation and release of DAMPs (alarmins). Mediators such as chymase released by mast cells, together with inflammatory cytokines (TNF-α and MIF) and chemokine signals (CXCL10), promote acute-phase protein responses (CRP, SAP and galectin-3), inflammatory amplification, and vascular endothelial destabilisation. At the central microvascular interface, activated and dysfunctional endothelial cells exhibit disrupted intercellular junctions and increased permeability. This structural barrier impairment facilitates plasma leakage via vascular permeability, local tissue oedema, and leukocyte transmigration into the extravascular (interstitial) spaces. Contemporaneously, acute-phase signalling in the liver and systemic compartments drive organ dysfunction and thrombocytopenia. To offset severe damage, counter-regulatory and repair signals (such as IL-1Ra and HGF) are mobilised. Overall, these intersecting biological cascades converge into a final, unified pathological state of vascular leakage and multi-organ dysfunction associated with clinical dengue disease progression. Created with BioRender.com. Larsson, M. (2026) https://BioRender.com/4hmjc8r. CRP, C-reactive protein; CXCL10: C-X-C motif chemokine ligand 10; DAMP, danger-associated molecular pattern; DENV, dengue virus; DWS-, dengue without warning signs; DWS+, dengue with warning signs; HGF, hepatocyte growth factor; IL-1β, interleukin-1 beta; IL-1Ra, interleukin-1 receptor antagonist; MCP-1, monocyte chemoattractant protein-1; MIF, macrophage migration inhibitory factor; SAP, serum amyloid P; TNF-α, tumour necrosis factor-alpha; TRAIL, TNF-related apoptosis-inducing ligand.

Our study has certain limitations that need to be acknowledged, such as the non-addressing of potential confounding factors including co-morbidities, as well as other flavivirus infections. Also, the plaque reduction neutralisation test for confirming DENV infection was not performed due to sample volume constraints. In addition, the cross-sectional nature of the study fails to establish a spatio-temporal relationship between the analytes across the varying severity of the disease. The study also did not provide data on the circulating DENV serotype. An important limitation was the unequal distribution between dengue patients and healthy controls, where all the healthy controls were male while dengue patients included both males and females, which likely impacts the inflammatory and immune mediators, representing a potential confounding factor. Although sex was considered in the adjusted analysis, the paucity of female healthy controls prevents complete exclusion of this confounding effect. Further, a relatively small number of patients in the severe dengue cohort restricted the statistical power for comparisons (involving severe dengue). Therefore, associations observed in this subgroup should be purely preliminary and requires due validation.

## 5 Conclusion

Our study has identified a coordinated endothelial dysfunction, heightened inflammatory responses, mast cell-derived mediators, and counter-regulatory immune pathways that collectively contribute to vascular leakage and organ injury across dengue severity. Of all the analytes measured, Ang-2, CXCL10, TRAIL, CRP, MIF, IL-1Ra, TNF-α, and chymase were strongly associated with disease severity, highlighting the potential involvement of endothelial activation and IFN-driven inflammation in dengue pathogenesis. Correlation analysis further indicated endothelial injury, as Ang-2 showed a strong positive association with thrombocytopenia and hepatic injury markers. Elevated chymase levels provides an association that warrants investigation of mast cell–driven vascular damage and potential mast cell–endothelial interactions leading to capillary leakage, while increased CRP reflects both acute‐phase responses as well as hepatic involvement. Overall, these findings identify several biomarkers linked to dengue severity. The statistical associations discussed here should nevertheless be interpreted as evidence of co-variation rather than direct molecular interactions, and these biological mechanisms need further validation. Addressing these gaps would require future studies that integrate comprehensive longitudinal sampling with expanded immunological profiling.

## Data Availability

The original contributions presented in the study are included in the article/supplementary material. Further inquiries can be directed to the corresponding authors.

## References

[1] Anshad AR Saravanan S Murugesan A Vighnesh R Raju S Kannan R . Aberrant systemic acute-phase complement responses in conjunction with soluble CR1 attribute to varying grades of dengue disease severity. Front Immunol. (2026) 16. doi: 10.3389/fimmu.2025.1731011 41624861 PMC12855062

[2] Halstead S Wilder-Smith A . Severe dengue in travellers: Pathogenesis, risk and clinical management. J Travel Med. (2019) 26:1–15. doi: 10.1093/jtm/taz062 31423536

[3] Nanaware N Banerjee A Mullick Bagchi S Bagchi P Mukherjee A . Dengue virus infection: A tale of viral exploitations and host responses. Viruses. (2021) 13:1967. doi: 10.3390/v13101967 34696397 PMC8541669

[4] Meena AA Murugesan A Sopnajothi S Yong YK Ganesh PS Vimali IJ . Increase of plasma TNF- α is associated with decreased levels of blood platelets in clinical dengue infection. Viral Immunol. (2020) 33:54–60. doi: 10.1089/vim.2019.0100 31532346

[5] Paz-Bailey G Adams LE Deen J Anderson KB Katzelnick LC . Dengue. Lancet. (2024) 403:667–82. doi: 10.1016/S0140-6736(23)02576-X 38280388 PMC12372472

[6] Narvaez F Montenegro C Juarez JG Zambrana JV Gonzalez K Videa E . Dengue severity by serotype and immune status in 19 years of pediatric clinical studies in Nicaragua. PloS Negl Trop Dis. (2025) 19:e0012811. doi: 10.1371/journal.pntd.0012811 39792951 PMC11750095

[7] Giang NT van Tong H Quyet D Hoan NX Nghia TH Nam NM . Complement protein levels and MBL2 polymorphisms are associated with dengue and disease severity. Sci Rep. (2020) 10:14923. doi: 10.1038/s41598-020-71947-2 32913345 PMC7484760

[8] Khanam A Gutiérrez-Barbosa H Lyke KE Chua JV . Immune-mediated pathogenesis in dengue virus infection. Viruses. (2022) 14:2575. doi: 10.3390/v14112575 36423184 PMC9699586

[9] Imad HA Phumratanaprapin W Phonrat B Chotivanich K Charunwatthana P Muangnoicharoen S . Cytokine expression in dengue fever and dengue hemorrhagic fever patients with bleeding and severe hepatitis. Am J Trop Med Hyg. (2020) 102:943–50. doi: 10.4269/ajtmh.19-0487 32124729 PMC7204576

[10] Goswami RP Mukherjee A Biswas T Karmakar PS Ghosh A . Two cases of dengue meningitis: A rare first presentation. J Infection Developing Countries. (2012) 6:208–11. doi: 10.3855/jidc.2241 22337854

[11] Vaidya A Salvi SP . Gastrointestinal and hepatic manifestations and outcome in dengue multi-organ dysfunction syndrome: data from a tertiary referral center. Infect Dis Trop Med. (2023) 9. doi: 10.32113/idtm_20239_1181

[12] Castilho BM Silva MT Freitas ARR Fulone I Lopes LC . Factors associated with thrombocytopenia in patients with dengue fever: a retrospective cohort study. BMJ Open. (2020) 10:e035120. doi: 10.1136/bmjopen-2019-035120 32928847 PMC7488788

[13] Dalrymple NA Mackow ER . Endothelial cells elicit immune-enhancing responses to dengue virus infection. J Virol. (2012) 86:6408–15. doi: 10.1128/JVI.00213-12 22496214 PMC3393559

[14] Cipitelli MC Paiva IA Badolato-Corrêa J Marinho CF Fiestas Solórzano VE da Costa Faria NR . Subsets of cytokines and chemokines from DENV-4-infected patients could regulate the endothelial integrity of cultured microvascular endothelial cells. Pathogens. (2022) 11:509. doi: 10.3390/pathogens11050509 35631030 PMC9144803

[15] Mishra H Ngai M Crowley VM Tran V Painaga MSS Gaite JY . The Angiopoietin–Tie-2 axis in children and young adults with dengue virus infection in the Philippines. Am J Trop Med Hyg. (2024) 111:887–96. doi: 10.4269/ajtmh.24-0115 39137755 PMC11448529

[16] Samanta J . Dengue and its effects on liver. World J Clin cases. (2015) 3:125. doi: 10.12998/wjcc.v3.i2.125 25685758 PMC4317605

[17] Brasier AR Ju H Garcia J Spratt HM Victor SS Forshey BM . A three-component biomarker panel for prediction of dengue hemorrhagic fever. Am J Trop Med Hyg. (2012) 86:341–8. doi: 10.4269/ajtmh.2012.11-0469 22302872 PMC3269290

[18] Conroy AL Gélvez M Hawkes M Rajwans N Tran V Liles WC . Host biomarkers are associated with progression to dengue haemorrhagic fever: a nested case-control study. Int J Infect Dis. (2015) 40:45–53. doi: 10.1016/j.ijid.2015.07.027 26255888

[19] Lai Y-C Chao C-H Yeh T-M . Roles of macrophage migration inhibitory factor in dengue pathogenesis: From pathogenic factor to therapeutic target. Microorganisms. (2020) 8:891. doi: 10.3390/microorganisms8060891 32545679 PMC7356240

[20] Mukherjee S Saha B Tripathi A . Clinical significance of differential serum-signatures for early prediction of severe dengue among Eastern Indian patients. Clin Exp Immunol. (2022) 208:72–82. doi: 10.1093/cei/uxac018 35348620 PMC9113256

[21] Gurugama P Jayarajah U Wanigasuriya K Wijewickrama A Perera J Seneviratne SL . Renal manifestations of dengue virus infections. J Clin Virol. (2018) 101:1–6. doi: 10.1016/j.jcv.2018.01.001 29414180

[22] Keshav LB K A Malhotra K Shetty S . Lung manifestation of dengue fever: A retrospective study. Cureus. (2024) 16:e60655. doi: 10.7759/cureus.60655 38903312 PMC11187467

[23] Mehmood A Afzal MW Ahmad M Mufti M Malik J Zaidi SMJ . Respiratory sequelae of dengue fever. Trop Doctor. (2023) 53:237–40. doi: 10.1177/00494755221127355 36579820

[24] Sánchez JL Salgado DM Vega MR Castro-Trujillo S Narváez CF . Utility of the WHO dengue guidelines in pediatric immunological studies. J Trop Pediatr. (2024) 70. doi: 10.1093/tropej/fmae014 39002166

[25] WHO . Dengue Guidelines for Diagnosis, Treatment, Prevention and Control Vol. 1. Geneva, Switzerland: WHO Press (2009). 23762963

[26] Mapalagamage M Handunnetti SM Wickremasinghe AR Premawansa G Thillainathan S Fernando T . High levels of serum angiopoietin 2 and angiopoietin 2/1 ratio at the critical stage of dengue hemorrhagic fever in patients and association with clinical and biochemical parameters. J Clin Microbiol. (2020) 58. doi: 10.1128/JCM.00436-19 31941693 PMC7098750

[27] Zhang Y Kontos CD Annex BH Popel AS . Angiopoietin-Tie signaling pathway in endothelial cells: A computational model. IScience. (2019) 20:497–511. doi: 10.1016/j.isci.2019.10.006 31655061 PMC6806670

[28] Mariko R Darwin E Yanwirasti Y Hadinegoro SR . The difference of angiopoietin-2 levels between dengue hemorrhagic fever patients with shock and without shock. Open Access Macedonian J Med Sci. (2019) 7:2119–22. doi: 10.3889/oamjms.2019.569 31456836 PMC6698111

[29] Michels M Van Der Ven AJAM Djamiatun K Fijnheer R De Groot PG Griffioen AW . Imbalance of angiopoietin-1 and angiopoetin-2 in severe dengue and relationship with thrombocytopenia, endothelial activation, and vascular stability. Am J Trop Med Hyg. (2012) 87:943–6. doi: 10.4269/ajtmh.2012.12-0020 22949515 PMC3516273

[30] Nor FM Khamis SN Wang SM Mokhtar MAM Yuhana MY Hoh BP . Evaluation of CXCL10 as potential biomarker for early detection of severe dengue. Int J Infect Dis. (2018) 73:395. doi: 10.1016/j.ijid.2018.04.431042574925

[31] Yong YK Tan HY Jen SH Shankar EM Natkunam SK Sathar J . Aberrant monocyte responses predict and characterize dengue virus infection in individuals with severe disease. J Transl Med. (2017) 15:121. doi: 10.1186/s12967-017-1226-4 28569153 PMC5452397

[32] Calderón-Peláez M-A Coronel-Ruiz C Castellanos JE Velandia-Romero ML . Endothelial dysfunction, HMGB1, and dengue: An enigma to solve. Viruses. (2022) 14:1765. doi: 10.3390/v14081765 36016387 PMC9414358

[33] Nwe KM Ngwe Tun MM Myat TW Sheng Ng CF Htun MM Lin H . Acute-phase serum cytokine levels and correlation with clinical outcomes in children and adults with primary and secondary dengue virus infection in Myanmar between 2017 and 2019. Pathogens. (2022) 11:558. doi: 10.3390/pathogens11050558 35631079 PMC9144711

[34] de-Oliveira-Pinto LM Gandini M Freitas LP Siqueira MM Marinho CF Setúbal S . Profile of circulating levels of IL-1Ra, CXCL10/IP-10, CCL4/MIP-1β and CCL2/MCP-1 in dengue fever and parvovirosis. Memórias Do Instituto Oswaldo Cruz. (2012) 107:48–56. doi: 10.1590/S0074-02762012000100007 22310535

[35] Gandini M Petitinga-Paiva F Marinho CF Correa G De Oliveira-Pinto LM Souza LJ . Dengue virus induces NK cell activation through TRAIL expression during infection. Mediators Inflammation. (2017) 2017:1–10. doi: 10.1155/2017/5649214 29038620 PMC5605866

[36] Limonta D Torrentes‐Carvalho A Marinho CF de Azeredo EL de Souza LJ Motta‐Castro ARC . Apoptotic mediators in patients with severe and non‐severe dengue from Brazil. J Med Virol. (2014) 86:1437–47. doi: 10.1002/jmv.23832 24170344 PMC7167172

[37] Becerra A Warke RV Martin K Xhaja K de Bosch N Rothman AL . Gene expression profiling of dengue infected human primary cells identifies secreted mediators *in vivo*. J Med Virol. (2009) 81:1403–11. doi: 10.1002/jmv.21538 19551822 PMC2893327

[38] Tramontini Gomes de Sousa Cardozo F Baimukanova G Lanteri MC Keating SM Moraes Ferreira F Heitman J . Serum from dengue virus-infected patients with and without plasma leakage differentially affects endothelial cells barrier function *in vitro*. PloS One. (2017) 12:e0178820. doi: 10.1371/journal.pone.0178820 28586397 PMC5460851

[39] de la Cruz Hernández SI Puerta-Guardo HN Flores Aguilar H González Mateos S López Martinez I Ortiz-Navarrete V . Primary dengue virus infections induce differential cytokine production in Mexican patients. Memórias Do Instituto Oswaldo Cruz. (2016) 111:161–7. doi: 10.1590/0074-02760150359 27008374 PMC4804498

[40] Gregorova M Santopaolo M Garner LC Hayati RF Diamond D Ramamurthy N . Early NK-cell and T-cell dysfunction marks progression to severe dengue in patients with obesity and healthy weight. Nat Commun. (2025) 16:5569. doi: 10.1038/s41467-025-60941-9 40595657 PMC12214611

[41] Rao R Nayak S Pandey A Kamath S . Diagnostic performance of C-reactive protein level and its role as a potential biomarker of severe dengue in adults. Asian Pacific J Trop Med. (2020) 13:358. doi: 10.4103/1995-7645.289440

[42] Kumar Y Liang C Bo Z Rajapakse JC Ooi EE Tannenbaum SR . Serum proteome and cytokine analysis in a longitudinal cohort of adults with primary dengue infection reveals predictive markers of DHF. PloS Negl Trop Dis. (2012) 6:e1887. doi: 10.1371/journal.pntd.0001887 23209847 PMC3510095

[43] Liu K-T Liu Y-H Chen Y-H Lin C-Y Huang C-H Yen M-C . Serum galectin-9 and galectin-3-binding protein in acute dengue virus infection. Int J Mol Sci. (2016) 17:832. doi: 10.3390/ijms17060832 27240351 PMC4926366

[44] Vuong NL Le Duyen HT Lam PK Tam DTH Vinh Chau NV Van Kinh N . C-reactive protein as a potential biomarker for disease progression in dengue: a multi-country observational study. BMC Med. (2020) 18:35. doi: 10.1186/s12916-020-1496-1 32063229 PMC7025413

[45] Rathore APS Senanayake M Athapathu AS Gunasena S Karunaratna I Leong WY . Serum chymase levels correlate with severe dengue warning signs and clinical fluid accumulation in hospitalized pediatric patients. Sci Rep. (2020) 10:11856. doi: 10.1038/s41598-020-68844-z 32678248 PMC7367272

[46] Tissera H Rathore APS Leong WY Pike BL Warkentien TE Farouk FS . Chymase level is a predictive biomarker of dengue hemorrhagic fever in pediatric and adult patients. J Infect Dis. (2017) 216:1112–21. doi: 10.1093/infdis/jix447 28968807 PMC5853622

[47] Sherif NA Zayan AH Elkady AH Ghozy S Ahmed AR Omran ES . Mast cell mediators in relation to dengue severity: A systematic review and meta‐analysis. Rev Med Virol. (2020) 30. doi: 10.1002/rmv.2084 31709696

[48] Teo A Le CTT Tan T Chia PY Yeo TW . Febrile phase soluble urokinase plasminogen activator receptor and olfactomedin 4 as prognostic biomarkers for severe dengue in adults. Clin Infect Dis. (2024) 78:788–96. doi: 10.1093/cid/ciad637 37823481

[49] McBride A Duyen HTL Vuong NL Tho PV Tai LTH Phong NT . Endothelial and inflammatory pathophysiology in dengue shock: New insights from a prospective cohort study in Vietnam. PloS Negl Trop Dis. (2024) 18:e0012071. doi: 10.1371/journal.pntd.0012071 38536887 PMC11020502

